# Supplementary material for: Changes in Spirulina’s Physical and Chemical Properties during Submerged and Solid-State Lacto-Fermentation
Source: Toxins (Basel). 2023 Jan 13;15(1):75. doi: 10.3390/toxins15010075 (PMC9862786; doi:10.3390/toxins15010075)
Supplement: Supplementary file 1 [file toxins-15-00075-s001.zip › toxins-2139489-supplementary.pdf]

Supplementary Material

Supplementary tables with experimental results

Table S1 Changes in the pH values and color coordinates (L*, a* and b*) in the Spirulina samples.

Spirulina samples	Fermentation		Color coordinates, NBS			pH
	Duration, h	Conditions	L*	a*	b*	
**Control (I)**	-		23.4±0.4^{b}	0.801±0.031^{b}	2.25±0.02^{b}	6.85±0.02^{b}
**Control (II)**	-		22.5±0.1^{a}	0.440±0.006^{a}	1.55±0.02^{a}	6.33±0.03^{a}
***Lactiplantibacillus plantarum* No. 122**	24h	SMF	23.3±0.2^{aB}	0.520±0.004^{aC}	2.01±0.01^{aB}	4.97±0.03^{bC}
	48h		23.5±0.1^{aB}	0.620±0.021^{bD}	2.08±0.03^{bC}	4.89±0.02^{aB}
	24h	SSF	24.5±0.4^{bC}	0.052±0.001^{aA}	2.07±0.06^{bB}	4.81±0.02^{aA}
	48h		22.4±0.2^{aA}	0.480±0.003^{bB}	1.75±0.01^{aA}	4.79±0.02^{aA}
***Lacticaseibacillus casei* No. 210**	24h	SMF	23.7±0.4^{aC}	0.163±0.035^{aB}	2.07±0.02^{aC}	4.80±0.01^{bB}
	48h		23.3±0.3^{aC}	0.330±0.042^{bC}	2.13±0.01^{bD}	4.75±0.03^{aA}
	24h	SSF	21.1±0.1^{aA}	0.044±0.004^{aA}	1.84±0.01^{aA}	4.82±0.03^{bB}
	48h		22.1±0.3^{bB}	0.141±0.010^{bB}	1.97±0.03^{bB}	4.72±0.05^{aA}
***Lactobacillus curvatus* No. 51**	24h	SMF	23.0±0.1^{aB}	0.641±0.009^{aC}	2.01±0.02^{aB}	5.19±0.02^{bC}
	48h		22.9±0.2^{aB}	0.682±0.024^{bD}	2.07±0.03^{bC}	4.86±0.01^{aA}
	24h	SSF	21.0±0.1^{aA}	0.311±0.005^{bB}	1.89±0.05^{aA}	5.19±0.04^{bC}
	48h		24.6±0.2^{bC}	0.053±0.004^{aA}	2.37±0.02^{bD}	5.05±0.06^{aB}
***Lacticaseibacillus paracasei* No. 244**	24h	SMF	24.0±0.1^{bC}	1.08±0.02^{aC}	2.08±0.01^{aB}	5.44±0.02^{aC}
	48h		23.1±0.4^{aB}	1.39±0.11^{bD}	2.13±0.01^{bC}	5.69±0.01^{bD}
	24h	SSF	21.1±0.2^{aA}	0.340±0.011^{bB}	1.74±0.01^{aA}	5.34±0.02^{bB}
	48h		21.7±0.1^{bB}	-0.070±0.001^{aA}	2.24±0.02^{bD}	5.08±0.03^{aA}
***Lactobacillus coryniformis* No. 71**	24h	SMF	22.9±0.3^{aA}	0.310±0.005^{aC}	2.08±0.02^{aB}	4.98±0.01^{bC}
	48h		23.0±0.3^{aA}	0.552±0.021^{bD}	2.07±0.01^{aB}	4.75±0.03^{aA}
	24h	SSF	22.8±0.3^{aA}	0.272±0.002^{bB}	1.93±0.03^{aA}	5.11±0.05^{bD}
	48h		22.4±0.4^{aA}	0.000±0.001^{aA}	2.21±0.04^{bC}	4.92±0.02^{aB}
***Pediococcus pentosaceus* No. 183**	24h	SMF	23.1±0.3^{aC}	0.554±0.037^{aC}	1.97±0.02^{aB}	4.85±0.04^{aB}
	48h		23.3±0.1^{aC}	0.841±0.052^{bD}	2.06±0.03^{bC}	4.81±0.02^{aB}
	24h	SSF	22.4±0.1^{bB}	0.141±0.002^{aA}	2.00±0.02^{bB}	4.54±0.01^{aA}
	48h		21.3±0.2^{aA}	0.412±0.001^{bB}	1.86±0.06^{aA}	4.79±0.02^{bB}
***Levilactobacillus brevis* No. 173**	24h	SMF	23.6±0.2^{bC}	0.068±0.008^{aA}	2.35±0.05^{bC}	4.95±0.03^{aB}
	48h		23.1±0.1^{aB}	1.35±0.01^{bD}	1.78±0.03^{aA}	4.98±0.01^{aB}
	24h	SSF	22.8±0.3^{bB}	0.133±0.001^{aB}	2.04±0.05^{bB}	5.20±0.03^{bC}
	48h		21.5±0.1^{aA}	0.422±0.003^{bC}	1.81±0.01^{aA}	4.10±0.02^{aA}
***Pediococcus acidilactici* No. 29**	24h	SMF	22.7±0.1^{aB}	0.427±0.013^{aC}	2.08±0.02^{bB}	4.97±0.02^{bB}
	48h		23.0±0.3^{aB}	0.926±0.009^{bD}	1.90±0.01^{aA}	4.82±0.03^{aA}
	24h	SSF	23.4±0.2^{bB}	0.051±0.002^{aA}	2.06±0.02^{aB}	4.84±0.04^{aA}

	48h		22.3±0.2^{aA}	0.270±0.002^{bB}	2.08±0.03^{aB}	4.79±0.02^{aA}
***Leuconostoc mesenteroides* No. 225**	24h	SMF	22.7±0.3^{aC}	0.478±0.006^{aC}	2.13±0.02^{bB}	4.91±0.03^{bB}
	48h		23.0±0.1^{aC}	0.696±0.004^{bD}	1.97±0.01^{aA}	4.69±0.01^{aA}
	24h	SSF	22.2±0.1^{bB}	0.253±0.001^{bB}	2.09±0.03^{aB}	5.20±0.06^{bC}
	48h		21.0±0.1^{aA}	-0.180±0.001^{aA}	2.13±0.02^{aB}	4.93±0.01^{aB}
***Liquorilactobacillus uvarum* No. 245**	24h	SMF	22.8±0.2^{aB}	0.403±0.011^{aB}	2.11±0.03^{bC}	4.88±0.02^{aB}
	48h		23.1±0.2^{aB}	1.07±0.21^{bD}	1.88±0.04^{aB}	4.97±0.01^{bC}
	24h	SSF	23.2±0.1^{bB}	0.211±0.002^{aA}	2.15±0.01^{bC}	4.77±0.03^{aA}
	48h		19.2±0.2^{aA}	0.552±0.003^{bC}	1.73±0.03^{aA}	4.75±0.02^{aA}

Control (I) - Spirulina powder and water mixture, 1:20 w/w; Control (II) - Spirulina powder and water mixture, 1:2 w/w; SSF – solid state fermentation; SMF – submerged fermentation; L* - lightness; a* - redness or −a* - greenness; b* - yellowness or −b* - blueness; NBS - National Bureau of Standards units. Data are represented as means (n = 3) ± standard error. $^{a\text{-}b}$ mean values denoted with different letters, indicates significantly different values between the different duration (24 and 48 h) of fermentation ($p \leq 0.05$); $^{A\text{-}D}$ mean values denoted with different letters, indicates significantly different values between samples fermented with the same strain at different conditions ($p \leq 0.05$).

Table S2. L-Glutamic acid (L-Glu) and gamma-aminobutyric acid (GABA) concentration in the spirulina samples.

Spirulina samples	Fermentation		Gamma-aminobutyric acid, mg/kg	L- Glutamic acid, mg/kg
	Duration, h	Conditions		
**Control (I)**	-		2.01±0.009^{a}	242.2±8.789^{a}
**Control (II)**	-		17.2±0.231^{b}	2296.4±11.325^{b}
***Lactiplantibacillus plantarum* No. 122**	24h	SMF	54.4±1.431^{aA}	221.5±2.114^{aA}
	48h		89.3±2.211^{bB}	300.1±2.952^{bB}
	24h	SSF	161.7±8.519^{aC}	4062.1±9.997^{bD}
	48h		228.6±9.008^{bD}	3032.8±10.821^{aC}
***Lacticaseibacillus casei* No. 210**	24h	SMF	17.4±0.574^{aA}	255.4±9.322^{aA}
	48h		25.3±0.971^{bB}	486.9±8.124^{bB}
	24h	SSF	27.6±1.641^{aC}	2781.2±12.220^{aC}
	48h		37.0±0.992^{bD}	3296.0±11.346^{bD}
***Lactobacillus curvatus* No. 51**	24h	SMF	8.94±0.182^{aA}	283.2±9.102^{aA}
	48h		17.1±0.452^{bB}	270.0±4.358^{aA}
	24h	SSF	19.3±0.517^{aC}	3106.3±9.781^{aB}
	48h		41.1±3.031^{bD}	5506.4±18.347^{bC}
***Lacticaseibacillus paracasei* No. 244**	24h	SMF	212.6±4.037^{aA}	15.1±1.287^{aA}
	48h		286.5±3.015^{bB}	41.7±3.217^{bB}
	24h	SSF	2016.4±10.354^{aC}	1783.8±3.267^{bD}
	48h		2395.9±12.992^{bD}	572.4±4.211^{aC}
***Lactobacillus coryniformis* No. 71**	24h	SMF	13.1±0.311^{aA}	260.9±2.142^{aA}
	48h		62.7±2.214^{bD}	435.4±3.001^{bB}
	24h	SSF	25.4±3.613^{aB}	2914.2±9.886^{aC}
	48h		34.6±2.097^{bC}	3569.7±10.287^{bD}
***Pediococcus pentosaceus* No. 183**	24h	SMF	31.1±2.781^{aA}	331.1±3.521^{aA}
	48h		34.8±1.024^{aA}	417.7±3.441^{bB}
	24h	SSF	28.6±1.082^{aA}	2751.6±12.346^{aC}
	48h		53.1±3.112^{bB}	2896.1±14.952^{bD}
***Levilactobacillus brevis* No. 173**	24h	SMF	12.0±0.219^{aA}	356.6±3.621^{bB}
	48h		187.3±10.301^{bD}	33.2±1.067^{aA}
	24h	SSF	22.6±0.879^{aB}	3302.4±9.889^{aC}
	48h		58.8±1.257^{bC}	3841.1±10.025^{bD}
***Pediococcus acidilactici* No. 29**	24h	SMF	19.9±0.932^{aA}	287.8±2.077^{aA}
	48h		53.7±2.057^{bB}	303.4±4.202^{bB}
	24h	SSF	89.4±2.345^{bD}	3018.3±12.221^{aC}
	48h		82.6±1.966^{aC}	4271.9±14.167^{bD}
***Leuconostoc mesenteroides* No. 225**	24h	SMF	169.5±9.001^{aA}	23.4±3.945^{aA}
	48h		162.1±10.147^{aA}	28.6±4.027^{aA}
	24h	SSF	1264.4±13.456^{bC}	2596.9±11.367^{aB}

	48h		199.7±8.034^{aB}	3209.4±12.956^{bC}
***Liquorilactobacillus uvarum* No. 245**	24h	SMF	53.6±3.078^{aA}	355.6±8.645^{bB}
	48h		224.6±11.214^{bC}	280.1±7.021^{aA}
	24h	SSF	217.4±8.894^{bC}	2621.2±18.361^{aC}
	48h		164.8±10.092^{aB}	2908.3±19.993^{bD}

Control (I) - Spirulina powder and water mixture, 1:20 w/w; Control (II) - Spirulina powder and water mixture, 1:2 w/w; SSF – solid state fermentation; SMF – submerged fermentation; Data are represented as means (n = 3) ± standard error. $^{a-b}$ mean values denoted with different letters, indicates significantly different values between the different duration (24 and 48 h) of fermentation ($p \leq 0.05$); $^{A-D}$ mean values denoted with different letters, indicates significantly different values between samples fermented with the same strain at different conditions ($p \leq 0.05$).

Table S3. Biogenic amine (BA) content (mg/kg) in the Spirulina samples.

Spirulina samples	Fermentation		Biogenic amines concentration, mg/kg							
	Duration, h	Conditions	TRP	PHE	PUT	CAD	HIS	TYR	SPRMD	SPRM
**Control (I)**	-		2.32±0.99^{a}	nd	5.43±1.05^{a}	0.873±0.098^{a}	nd	6.32±1.13^{a}	202.3±11.2^{a}	11.6±3.2^{a}
**Control (II)**	-		2.66/1.02^{a}	nd	6.79±1.11^{a}	0.939±0.102^{a}	nd	7.78±1.23^{a}	279.1±12.8^{b}	13.8±4.1^{a}
Lactiplantibacillus plantarum **No. 122**	24h	SMF	nd	nd	67.7±4.3^{aA}	nd	nd	nd	50.8±4.1a^{A}	nd
	48h	SMF	nd	nd	84.0±5.2^{bB}	nd	nd	3.53±0.99^{A}	52.7±4.3a^{A}	nd
	24h	SSF	6.08±0.72^{a}	nd	74.0±5.6^{aA}	10.0±1.2	nd	23.4±1.3^{aB}	458.9±15.6^{aB}	19.6±1.2^{a}
	48h	SSF	5.86±0.95^{a}	nd	681.3±15.4^{bC}	nd	nd	26.6±2.5^{bB}	473.6±17.9^{aB}	20.2±1.4^{a}
Lacticaseibacillus casei **No. 210**	24h	SMF	nd	nd	20.5±1.7^{aA}	nd	nd	3.55±0.63^{bB}	52.8±3.8^{aA}	nd
	48h	SMF	nd	nd	77.2±4.9^{bC}	nd	nd	1.19±0.71^{aA}	57.2±4.2^{aA}	nd
	24h	SSF	5.76±1.00^{a}	nd	34.7±2.2^{aB}	33.2±3.1	8.54±0.39	32.0±2.4^{bD}	358.1±15.0^{aB}	35.8±2.1^{b}
	48h	SSF	4.68±0.53^{a}	nd	117.9±16.5^{bD}	nd	nd	24.4±2.1^{aC}	466.2±14.8^{bC}	19.4±1.1^{a}
Lactobacillus curvatus **No. 51**	24h	SMF	nd	nd	94.3±6.4^{bB}	nd	nd	2.18±0.12^{A}	59.0±4.4^{bB}	nd
	48h	SMF	nd	nd	71.8±3.2^{aA}	nd	nd	nd	46.7±3.4^{aA}	nd
	24h	SSF	2.66±0.32^{a}	nd	755.7±16.9^{aC}	nd	nd	21.8±2.5^{aB}	524.9±15.6^{bD}	18.1±1.6^{a}
	48h	SSF	5.36±0.61^{b}	nd	789.3±15.7^{bD}	nd	nd	25.1±2.2^{aB}	476.0±15.7^{aC}	19.3±1.2^{a}
Lacticaseibacillus paracasei **No. 244**	24h	SMF	4.77±0.8^{A}	nd	5.84±0.97^{aA}	nd	nd	45.2±2.1^{aA}	51.1±3.9^{aA}	nd
	48h	SMF	nd	nd	274.9±19.8^{bB}	nd	nd	108.9±16.7^{bB}	61.6±4.7^{bB}	nd
	24h	SSF	4.56±0.65^{aA}	nd	730.4±14.9^{aC}	2.24±0.43^{a}	32.1±1.9^{a}	167.7±15.3^{aC}	487.4±14.9^{aC}	18.6±0.9^{a}
	48h	SSF	6.12±0.99^{aA}	nd	814.8±16.8^{bD}	24.4±1.3^{b}	137.9±5.2^{b}	609.4±15.9^{bD}	458.6±15.1^{aC}	18.3±1.0^{a}
Lactobacillus coryniformis **No. 71**	24h	SMF	nd	nd	70.1±6.4a^{A}	nd	nd	1.50±0.23^{aA}	48.1±3.2^{aA}	nd
	48h	SMF	nd	nd	81.0±4.6a^{A}	nd	nd	9.41±0.97^{bB}	54.3±4.2^{aA}	nd
	24h	SSF	3.30±0.64^{a}	nd	705.3±15.1^{aB}	nd	nd	32.6±3.1^{aC}	467.2±16.0^{aB}	17.2±1.4^{a}
	48h	SSF	3.32±0.96^{a}	nd	736.9±16.9^{aB}	nd	nd	43.1±2.9^{bD}	486.6±14.3^{aB}	19.9±1.7^{a}
Pediococcus pentosaceus **No. 183**	24h	SMF	nd	nd	nd	nd	nd	nd	41.3±3.1^{aA}	nd
	48h	SMF	nd	nd	86.6±3.9^{C}	nd	nd	1.98±1.8^{A}	59.7±3.9^{Bb}	nd
	24h	SSF	4.59±0.47^{a}	nd	20.8±1.6^{aA}	6.32±1.01	nd	30.7±2.1^{aB}	469.4±14.9^{aC}	18.6±2.1^{a}
	48h	SSF	5.71±1.01^{a}	nd	32.4±3.7^{bB}	nd	nd	34.3±3.0^{aB}	492.4±15.5^{aC}	19.6±2.2^{a}
Levilactobacillus brevis **No. 173**	24h	SMF	nd	nd	92.3±7.1^{aA}	nd	nd	nd	53.9±4.3^{aA}	nd
	48h	SMF	nd	nd	95.7±4.2^{aA}	nd	1.32±0.41	28.3±2.1^{A}	54.4±4.0^{aA}	nd
	24h	SSF	5.84±0.86^{a}	nd	833.4±17.0^{aB}	nd	nd	26.6±2.4^{aA}	516.2±15.7^{aB}	19.2±2.4^{a}
	48h	SSF	5.55±0.79^{a}	nd	854.7±16.2^{aB}	nd	nd	31.1±2.8^{aA}	518.7±15.6^{aB}	21.5±2.1^{a}
Pediococcus acidilactici **No. 29**	24h	SMF	nd	nd	77.3±6.5^{aA}	nd	nd	1.24±0.36^{aA}	50.4±3.3^{aA}	nd
	48h	SMF	nd	nd	91.1±5.3^{bB}	nd	nd	2.36±0.87^{aA}	57.5±4.2^{aA}	1.06±0.03^{A}
	24h	SSF	6.90±0.11^{a}	nd	798.6±16.4^{aC}	nd	nd	20.7±1.9^{aB}	489.2±14.9^{aB}	19.5±1.2^{aB}
	48h	SSF	6.60±0.36^{a}	nd	833.3±17.2^{bD}	nd	nd	22.4±2.1^{aB}	501.9±14.8^{aB}	20.3±2.4^{aB}
Leuconostoc mesenteroides **No. 225**	24h	SMF	nd	nd	96.1±4.7^{aA}	nd	nd	5.50±0.96^{aA}	49.7±3.5^{aA}	nd
	48h	SMF	nd	nd	99.9±4.9^{aA}	nd	nd	34.7±2.3^{bB}	52.4±4.6^{aA}	nd
	24h	SSF	5.06±0.88^{a}	nd	778.9±14.6^{aB}	1.93±0.97	1.78±0.82	85.4±3.8^{bD}	460.2±15.3^{aB}	18.9±2.1^{b}
	48h	SSF	5.32±0.86^{a}	nd	784.5±18.1^{aB}	nd	nd	61.2±3.0^{aC}	468.9±13.9^{aB}	8.21±0.96^{a}

***Liquorilactobacillus uvarum* No. 245**	24h	SMF	nd	nd	58.1±3.8^{aA}	nd	nd	0.99±0.09^{aA}	49.9±3.4^{aA}	nd
	48h		nd	nd	99.8±6.4^{bB}	nd	nd	17.7±1.4^{bB}	61.2±4.7^{bB}	1.52±0.08^{A}
	24h	SSF	6.35±0.75^{a}	nd	229.5±14.3^{aC}	nd	nd	22.0±1.2^{aC}	440.0±14.2^{aC}	17.2±1.8^{aB}
	48h		7.53±1.12^{a}	nd	670.1±15.2^{bD}	nd	nd	25.2±2.1^{aC}	457.9±14.4^{aC}	18.1±1.7^{aB}

Control (I) - Spirulina powder and water mixture, 1:20 w/w; Control (II) - Spirulina powder and water mixture, 1:2 w/w; SSF – solid state fermentation; SMF – submerged fermentation; TRP – tryptamine; PHE - phenylethylamine; PUT - putrescine; CAD - cadaverine; HIS - histamine; TYR - tyramine; SPRMD - spermidine; SPRM – spermine; nd - not determined.

Data are represented as means (n = 3) ± standard error. $^{a\text{-}b}$ mean values denoted with different letters, indicates significantly different values between the different duration (24 and 48 h) of fermentation ($p \leq 0.05$); $^{A\text{-}D}$ mean values denoted with different letters, indicates significantly different values between samples fermented with the same strain at different conditions ($p \leq 0.05$).

Table S4. Influence of the analyzed factors and their interaction on biogenic amine (BA) concentration in the Spirulina samples.

Factors and their interaction	Dependent Variable	p
Lactic acid bacteria strain used for fermentation	TRP	0.0001
	PUT	0.0001
	CAD	0.0001
	HIS	0.0001
	TYR	0.0001
	SPRMD	0.0001
	SPRM	0.0001
Duration of fermentation	TRP	.960
	PUT	0.0001
	CAD	0.0001
	HIS	0.0001
	TYR	0.0001
	SPRMD	0.0001
	SPRM	0.002
Conditions of fermentation (submerged or solid state)	TRP	0.0001
	PUT	0.0001
	CAD	0.0001
	HIS	0.0001
	TYR	0.0001
	SPRMD	0.0001
	SPRM	0.0001
Lactic acid bacteria strain used for fermentation * Duration of fermentation	TRP	0.0001
	PUT	0.0001
	CAD	0.0001
	HIS	0.0001
	TYR	0.0001
	SPRMD	0.0001
	SPRM	0.0001
Lactic acid bacteria strain used for fermentation * Conditions of fermentation (submerged or solid state)	TRP	0.0001
	PUT	0.0001
	CAD	0.0001
	HIS	0.0001
	TYR	0.0001
	SPRMD	0.0001
	SPRM	0.0001
Duration of fermentation * Conditions of fermentation (submerged or solid state)	TRP	0.0001
	PUT	0.0001
	CAD	0.0001
	HIS	0.0001
	TYR	0.0001
	SPRMD	.071
	SPRM	0.0001
Lactic acid bacteria strain used for fermentation * Duration of fermentation * Conditions of fermentation (submerged or solid state)	TRP	0.0001
	PUT	0.0001
	CAD	0.0001
	HIS	0.0001
	TYR	0.0001
	SPRMD	0.0001
	SPRM	0.0001

TRP – tryptamine; PUT - putrescine; CAD - cadaverine; HIS - histamine; TYR - tyramine; SPRMD - spermidine; SPRM – spermine. Influence of factor or factors interaction is recognised as statistically significant, when $p \leq 0.05$.

Table S5. Fatty acid (FA) profile in the Spirulina samples.

Spirulina samples	Fermentation		Fatty acid content, % from total fat content						
	Duration, h	Conditions	C16:0	C16:1	C18:0	C18:1 *cis, trans*	C18:2	C18:3 γ	C18:3 α
**Control (I)**	-		42.2±0.2a	3.61±0.02a	5.26±0.11b	2.47±0.01a	18.7±0.1a	27.8±0.1b	nd
**Control (II)**	-		51.6±0.5b	3.73±0.07b	2.67±0.02a	3.31±0.01b	23.5±0.2b	15.2±0.3a	nd
***Lactiplantibacillus plantarum* No. 122**	24h	SMF	43.0±0.1^{aA}	3.73±0.01^{bC}	4.54±0.03^{aC}	3.05±0.01^{aA}	17.3±0.2^{bB}	28.4±0.2^{aC}	nd
	48h		43.1±0.1^{aA}	3.35±0.06^{aA}	5.02±0.05^{bD}	3.45±0.03^{bD}	16.8±0.0^{aA}	28.3±0.1^{aC}	nd
	24h	SSF	53.6±0.2^{bC}	3.76±0.03^{bC}	3.18±0.01^{aA}	3.16±0.02^{aB}	19.7±0.1^{bD}	16.6±0.1^{aA}	nd
	48h		52.1±0.4^{aB}	3.57±0.02^{aB}	4.25±0.05^{bB}	3.33±0.02^{bC}	18.2±0.2^{aC}	17.9±0.2^{bB}	0.605±0.011
***Lacticaseibacillus casei* No. 210**	24h	SMF	42.4±0.3^{aA}	3.62±0.03^{aB}	4.56±0.09^{aB}	2.97±0.01^{aA}	17.5±0.1^{bB}	29.0±0.1^{aC}	nd
	48h		42.4±0.1^{aA}	3.68±0.11^{aB}	4.71±0.01^{bC}	3.17±0.02^{bB}	17.0±0.1^{aA}	29.1±0.3^{aC}	nd
	24h	SSF	50.5±0.6^{aB}	3.60±0.03^{bB}	3.74±0.01^{aA}	3.29±0.03^{aC}	21.1±0.4^{bD}	17.7±0.1^{aA}	nd
	48h		50.3±0.1^{aB}	3.49±0.01^{aA}	3.76±0.03^{aA}	3.37±0.01^{bD}	20.4±0.1^{aC}	18.1±0.1^{bB}	0,589±0.010
***Lactobacillus curvatus* No. 51**	24h	SMF	45.0±0.2^{aA}	2.92±0.04^{aA}	4.59±0.02^{aC}	4.19±0.06^{bC}	16.5±0.2^{aA}	26.8±0.4^{aC}	nd
	48h		44.8±0.2^{aA}	3.38±0.07^{bB}	5.77±0.01^{bD}	3.15±0.04^{aA}	16.3±0.0^{aA}	26.6±0.2^{aC}	nd
	24h	SSF	53.8±0.2^{aB}	3.75±0.01^{bD}	3.66±0.01^{aA}	3.10±0.02^{aA}	19.4±0.1^{aB}	16.4±0.2^{bB}	nd
	48h		54.0±0.4^{aB}	3.54±0.06^{aC}	3.91±0.02^{bB}	3.29±0.04^{bB}	19.3±0.3^{aB}	15.5±0.1^{aA}	0.513±0.002
***Lacticaseibacillus paracasei* No. 244**	24h	SMF	45.0±0.1^{aA}	3.29±0.01^{aA}	5.25±0.02^{aC}	3.00±0.01^{aA}	17.5±0.2^{aA}	26.0±0.1^{bD}	nd
	48h		44.6±0.3^{aA}	3.18±0.12^{aA}	5.19±0.08^{aC}	3.24±0.12^{bB}	18.3±0.1^{bB}	25.5±0.1^{aC}	nd
	24h	SSF	56.2±0.3^{aB}	3.54±0.02^{aB}	3.64±0.03^{aA}	3.22±0.03^{bB}	18.8±0.1^{bC}	14.6±0.1^{bB}	nd
	48h		56.6±0.4^{aB}	3.58±0.02^{aB}	3.73±0.03^{bB}	3.02±0.01^{aA}	18.2±0.2^{aB}	14.4±0.0^{aA}	0.523±0.001
***Lactobacillus coryniformis* No. 71**	24h	SMF	43.6±0.2^{aA}	3.57±0.01^{aA}	5.12±0.01^{aC}	3.14±0.01^{aA}	17.1±0.1^{aB}	27.5±0.3^{aC}	nd
	48h		43.1±0.3^{aA}	3.74±0.03^{bC}	5.40±0.04^{bD}	3.18±0.06^{aA}	16.9±0.2^{aB}	27.7±0.2^{aC}	nd
	24h	SSF	52.0±0.2^{aB}	3.76±0.03^{bC}	3.11±0.01^{bB}	3.17±0.02^{aA}	20.7±0.1^{bC}	16.8±0.1^{bB}	0.450±0.021
	48h		60.8±0.7^{bC}	3.68±0.01^{aB}	1.47±0.01^{aA}	3.81±0.01^{bB}	16.3±0.3^{aA}	14.0±0.1^{aA}	nd
***Pediococcus pentosaceus* No. 183**	24h	SMF	42.8±0.2^{aA}	3.58±0.03^{aA}	5.02±0.01^{bD}	3.44±0.02^{aA}	17.0±0.1^{aA}	28.2±0.1^{aC}	nd
	48h		42.7±0.1^{aA}	3.80±0.05^{bC}	4.98±0.01^{aC}	3.13±0.03^{aA}	17.0±0.1^{aA}	28.4±0.1^{aC}	nd
	24h	SSF	52.5±0.1^{aB}	3.69±0.05^{aB}	2.78±0.04^{aA}	2.98±0.01^{aB}	20.0±0.1^{aB}	18.1±0.2^{bB}	nd
	48h		52.0±0.2^{aB}	3.73±0.01^{aB}	3.66±0.05^{bB}	3.15±0.05^{bC}	20.4±0.2^{bC}	16.6±0.0^{aA}	0.418±0.009
***Levilactobacillus brevis* No. 173**	24h	SMF	44.1±0.1^{aA}	3.65±0.03^{aA}	4.87±0.06^{bC}	2.85±0.01^{bB}	17.4±0.1^{bD}	27.1±0.3^{bD}	nd
	48h		44.5±0.2^{bB}	3.61±0.01^{aA}	5.85±0.03^{aD}	3.06±0.01^{aA}	16.5±0.2^{aC}	26.5±0.1^{aC}	nd
	24h	SSF	53.1±0.1^{aC}	3.77±0.01^{bB}	3.65±0.04^{bB}	3.07±0.01^{aC}	19.2±0.2^{aB}	17.2±0.3^{bB}	nd
	48h		54.7±0.3^{bD}	3.60±0.03^{aA}	3.40±0.01^{aA}	3.17±0.02^{aC}	19.3±0.1^{aA}	15.3±0.1^{aA}	0.551±0.012
***Pediococcus acidilactici* No. 29**	24h	SMF	43.4±0.1^{aA}	3.72±0.11^{aA}	4.66±0.01^{aB}	3.18±0.08^{aA}	17.3±0.0^{bB}	27.7±0.2^{aB}	nd
	48h		43.7±0.3^{aA}	3.72±0.07^{aA}	5.01±0.05^{bC}	3.37±0.04^{bC}	16.4±0.1^{aA}	27.8±0.4^{aB}	nd
	24h	SSF	50.7±0.5^{aB}	3.64±0.01^{aA}	3.28±0.01^{aA}	3.21±0.03^{aA}	20.9±0.1^{aC}	17.9±0.1^{aA}	0.410±0.001^{a}
	48h		50.7±0.1^{aB}	3.73±0.02^{aA}	3.31±0.03^{aA}	3.28±0.01^{bB}	21.0±0.1^{bD}	17.5±0.3^{aA}	0.447±0.011^{b}
***Leuconostoc mesenteroides* No. 225**	24h	SMF	45.7±0.0^{aA}	3.03±0.01^{aA}	5.35±0.02^{bD}	3.97±0.05^{bC}	16.7±0.1^{aA}	25.3±0.2^{aC}	nd
	48h		45.8±0.1^{aA}	3.53±0.01^{bB}	4.96±0.03^{aC}	3.23±0.01^{aB}	16.9±0.2^{aA}	25.7±0.1^{bD}	nd
	24h	SSF	54.8±0.3^{aB}	3.47±0.05^{aB}	3.53±0.02^{bB}	3.21±0.04^{bB}	18.5±0.3^{aB}	15.9±0.1^{bB}	0.618±0.007^{b}

	48h		56.3 ± 0.4^{bC}	3.52 ± 0.01^{aB}	3.43 ± 0.01^{aA}	3.08 ± 0.01^{aA}	18.9 ± 0.1^{aB}	14.4 ± 0.2^{aA}	0.448 ± 0.004^{a}
***Liquorilactobacillus uvarum* No. 245**	24h	SMF	42.8 ± 0.1^{aA}	3.69 ± 0.01^{aB}	4.84 ± 0.07^{aC}	3.26 ± 0.03^{bC}	17.3 ± 0.3^{aA}	28.1 ± 0.1^{aC}	nd
	48h		43.3 ± 0.4^{aA}	3.64 ± 0.03^{aB}	4.81 ± 0.01^{aC}	3.13 ± 0.01^{aA}	16.9 ± 0.1^{aA}	28.1 ± 0.2^{aC}	nd
	24h	SSF	50.6 ± 0.1^{aB}	3.54 ± 0.06^{aA}	3.78 ± 0.01^{bB}	3.36 ± 0.03^{bD}	20.5 ± 0.4^{aB}	17.8 ± 0.0^{bB}	0.444 ± 0.013^{b}
	48h		51.5 ± 0.8^{aB}	3.66 ± 0.02^{bB}	3.26 ± 0.01^{aA}	3.20 ± 0.01^{aB}	20.9 ± 0.1^{aB}	17.1 ± 0.1^{aA}	0.399 ± 0.008^{a}

Control (I) - Spirulina powder and water mixture, 1:20 w/w; Control (II) - Spirulina powder and water mixture, 1:2 w/w; SSF – solid state fermentation; SMF – submerged fermentation; C16:0—methyl palmitate; C16:1—methyl palmitoleate; C18:0—methyl stearate; C18:1 *cis, trans - cis, trans*-9- oleic acid methyl ester; C18:2—methyl linoleate; C18:3ɣ—gamma- linolenic acid methyl ester; C18:3α—alfa linolenic acid methyl ester; nd - not determined. Data are represented as means (n = 3) ± standard error. $^{a-b}$ mean values denoted with different letters, indicates significantly different values between the different duration (24 and 48 h) of fermentation ($p \leq 0.05$); $^{A-D}$ mean values denoted with different letters, indicates significantly different values between samples fermented with the same strain at different conditions ($p \leq 0.05$).

Table S6. Influence of the analyzed factors and their interaction on fatty acid (FA) content in the Spirulina samples.

**Factors and their interaction**	**Dependent Variable**	***p***
**Lactic acid bacteria strain used for fermentation**	C16:0—methyl palmitate	0.0001
	C16:1—methyl palmitoleate	0.192
	C18:0—methyl stearate	0.029
	C18:1 *cis, trans* - *cis, trans*-9- oleic acid methyl ester	0.230
	C18:2—methyl linoleate	0.003
	C18:3γ—gamma- linolenic acid methyl ester	0.004
	C18:3α—alfa linolenic acid methyl ester	0.0001
**Duration of fermentation**	C16:0—methyl palmitate	0.084
	C16:1—methyl palmitoleate	0.433
	C18:0—methyl stearate	0.101
	C18:1 *cis, trans* - *cis, trans*-9- oleic acid methyl ester	0.448
	C18:2—methyl linoleate	0.464
	C18:3γ—gamma- linolenic acid methyl ester	0.286
	C18:3α—alfa linolenic acid methyl ester	0.0001
**Conditions of fermentation (submerged or solid state)**	C16:0—methyl palmitate	0.982
	C16:1—methyl palmitoleate	0.502
	C18:0—methyl stearate	0.0001
	C18:1 *cis, trans* - *cis, trans*-9- oleic acid methyl ester	0.372
	C18:2—methyl linoleate	0.038
	C18:3γ—gamma- linolenic acid methyl ester	0.864
	C18:3α—alfa linolenic acid methyl ester	0.0001
**Lactic acid bacteria strain used for fermentation * Duration of fermentation**	C16:0—methyl palmitate	0.001
	C16:1—methyl palmitoleate	0.338
	C18:0—methyl stearate	0.294
	C18:1 *cis, trans* - *cis, trans*-9- oleic acid methyl ester	0.042
	C18:2—methyl linoleate	0.0001
	C18:3γ—gamma- linolenic acid methyl ester	0.0001
	C18:3α—alfa linolenic acid methyl ester	0.0001
**Lactic acid bacteria strain used for fermentation * Conditions of fermentation (submerged or solid state)**	C16:0—methyl palmitate	0.001
	C16:1—methyl palmitoleate	0.604
	C18:0—methyl stearate	0.028
	C18:1 *cis, trans* - *cis, trans*-9- oleic acid methyl ester	0.263
	C18:2—methyl linoleate	0.001
	C18:3γ—gamma- linolenic acid methyl ester	0.001
	C18:3α—alfa linolenic acid methyl ester	0.0001
**Duration of fermentation * Conditions of fermentation (submerged or solid state)**	C16:0—methyl palmitate	0.101
	C16:1—methyl palmitoleate	0.529
	C18:0—methyl stearate	0.480
	C18:1 *cis, trans* - *cis, trans*-9- oleic acid methyl ester	0.426
	C18:2—methyl linoleate	0.177
	C18:3γ—gamma- linolenic acid methyl ester	0.621
	C18:3α—alfa linolenic acid methyl ester	0.0001
**Lactic acid bacteria strain used for fermentation * Duration of fermentation * Conditions of fermentation (submerged or solid state)**	C16:0—methyl palmitate	0.0001
	C16:1—methyl palmitoleate	0.929
	C18:0—methyl stearate	0.190
	C18:1 *cis, trans* - *cis, trans*-9- oleic acid methyl ester	0.206
	C18:2—methyl linoleate	0.0001
	C18:3γ—gamma- linolenic acid methyl ester	0.0001
	C18:3α—alfa linolenic acid methyl ester	0.0001

Influence of factor or factors interaction is recognised as statistically significant, when $p \leq 0.05$.

Table S7. Classification of fatty acids (FA) in the Spirulina samples.

**Spirulina samples**	**Fermentation**		**SFA**	**MUFA**	**PUFA**	**Omega-3**	**Omega-6**	**Omega-9**
	Duration, h	Conditions						
**Control (I)**	-		47.5±1.5^{a}	6.08±0.18^{a}	46.5±1.3^{b}	nd	46.5±1.4^{b}	6.08±0.18^{a}
**Control (II)**	-		54.2±1.2^{b}	7.04±0.21^{b}	38.7±1.2^{a}	nd	38.7±1.2^{a}	7.04±0.21^{b}
***Lactiplantibacillus plantarum* No. 122**	24h	SMF	47.5±1.4^{aA}	6.78±0.11^{aB}	45.7±1.1^{aB}	nd	45.7±1.1^{aB}	6.78±0.11^{aA}
	48h		48.1±0.9^{aA}	6.80±0.13^{aB}	45.1±1.3^{aB}	nd	45.1±1.0^{aB}	6.80±0.13^{aA}
	24h	SSF	56.8±1.3^{aB}	6.92±0.20^{aA}	36.3±1.1^{aA}	nd	36.3±1.3^{aA}	6.92±0.20^{aA}
	48h		56.4±1.8^{aB}	6.90±0.14^{aA}	36.7±1.0^{aA}	0.605±0.018	36.1±1.0^{aA}	6.98±0.14^{aA}
***Lacticaseibacillus casei* No. 210**	24h	SMF	47.0±1.4^{aA}	6.59±0.10^{aA}	46.5±1.2^{aB}	nd	46.5±1.6^{aB}	6.59±0.10^{aA}
	48h		47.1±0.9^{aA}	6.85±0.13^{bB}	46.1±1.4^{aB}	nd	46.1±1.4^{aB}	6.85±0.13^{bB}
	24h	SSF	54.2±1.1^{aB}	6.89±0.19^{aB}	38.8±1.3^{aA}	nd	38.8±1.2^{aA}	6.89±0.19^{aB}
	48h		54.1±1.5^{aB}	6.86±0.15^{aB}	39.1±1.2^{aA}	0.589±0.010	38.5±1.1^{aA}	6.86±0.15^{aB}
***Lactobacillus curvatus* No. 51**	24h	SMF	49.6±1.2^{aA}	7.11±0.17^{bB}	43.3±1.4^{aB}	nd	43.3±1.0^{aB}	7.11±0.17^{bB}
	48h		50.6±1.0^{aA}	6.53±0.09^{aA}	42.9±1.0^{aB}	nd	42.9±1.2^{aB}	6.53±0.09^{aA}
	24h	SSF	57.5±1.3^{aB}	6.85±0.13^{aB}	35.8±1.1^{aA}	nd	35.8±1.0^{aA}	6.85±0.13^{aB}
	48h		57.9±1.0^{aB}	6.83±0.19^{aB}	35.3±0.9^{aA}	0.513±0.014	34.8±1.1^{aA}	6.83±0.19^{aB}
***Lacticaseibacillus paracasei* No. 244**	24h	SMF	50.3±0.9^{aA}	6.29±0.10^{aA}	43.5±1.2^{aB}	nd	43.5±1.3^{aB}	6.29±0.29^{aA}
	48h		49.8±0.8^{aA}	6.42±0.15^{aA}	43.8±1.1^{aB}	nd	43.8±1.1^{aB}	6.42±0.15^{aA}
	24h	SSF	59.8±1.2^{aB}	6.76±0.12^{aA}	33.4±1.3^{aA}	nd	33.4±1.2^{aA}	6.76±0.12^{aA}
	48h		60.3±1.1^{aB}	6.60±0.11^{aA}	33.1±1.4^{aA}	0.523±0.012	32.6±1.1^{aA}	6.62±0.15^{aA}
***Lactobacillus coryniformis* No. 71**	24h	SMF	48.7±1.0^{aA}	6.71±0.08^{aA}	44.6±1.5^{aC}	nd	44.6±1.2^{aC}	6.71±0.08^{aA}
	48h		48.5±0.9^{aA}	6.92±0.10^{bB}	44.6±1.1^{aC}	nd	44.6±1.0^{aC}	6.92±0.12^{bB}
	24h	SSF	55.1±0.9^{aB}	6.93±0.21^{aB}	38.0±1.3^{bB}	0.450±0.009	37.5±1.2^{bB}	6.93±0.21^{aB}
	48h		62.3±1.2^{bC}	7.49±0.20^{bC}	30.3±0.9^{aA}	nd	30.3±1.0^{aA}	7.49±0.20^{bC}
***Pediococcus pentosaceus* No. 183**	24h	SMF	47.8±1.4^{aA}	7.02±0.19^{aA}	45.2±1.2^{aB}	nd	45.2±1.2^{aB}	7.02±0.19^{aA}
	48h		47.7±1.1^{aA}	6.93±0.14^{aA}	45.4±1.3^{aB}	nd	45.4±1.3^{aB}	6.93±0.14^{aA}
	24h	SSF	55.3±1.3^{aB}	6.67±0.13^{aA}	38.1±1.1^{bA}	nd	38.1±1.2^{aA}	6.67±0.13^{aA}
	48h		55.7±1.3^{aB}	6.88±0.11^{aA}	37.4±1.3^{aA}	0.418±0.011	37.0±1.3^{aA}	6.88±0.11^{aA}
***Levilactobacillus brevis* No. 173**	24h	SMF	49.0±1.2^{aA}	6.50±0.14^{aA}	44.5±1.2^{aB}	nd	44.5±1.1^{aB}	6.53±0.15^{aA}
	48h		50.4±0.9^{aA}	6.67±0.20^{aA}	43.0±1.2^{aB}	nd	43.0±1.0^{aB}	6.67±0.20^{aA}
	24h	SSF	56.8±0.4^{aB}	6.84±0.16^{aA}	36.4±1.4^{aA}	nd	36.4±1.0^{aA}	6.84±0.16^{aA}
	48h		58.1±1.5^{aB}	6.77±0.14^{aA}	35.1±1.2^{aA}	0.551±0.015	34.6±1.4^{aA}	6.77±0.14^{aA}
***Pediococcus acidilactici* No. 29**	24h	SMF	48.1±1.1^{aA}	6.90±0.12^{aA}	45.0±1.0^{aB}	nd	45.0±1.2^{aB}	6.91±0.11^{aA}
	48h		48.7±1.0^{aA}	7.09±0.21^{aA}	44.2±1.4^{aB}	nd	44.2±1.2^{aB}	7.09±0.21^{aA}
	24h	SSF	54.0±1.2^{aB}	6.85±0.20^{aA}	39.2±1.0^{aA}	0.410±0.008^{a}	38.8±1.1^{aA}	6.85±0.21^{aA}
	48h		54.0±0.9^{aB}	7.01±0.13^{aA}	38.9±1.5^{aA}	0.447±0.009^{b}	38.5±1.3^{aA}	7.01±0.14^{aA}
***Leuconostoc mesenteroides* No. 225**	24h	SMF	51.1±0.7^{aA}	7.00±0.15^{aA}	42.3±1.3^{aB}	nd	42.0±1.1^{aB}	7.00±0.14^{aA}
	48h		50.8±1.3^{aA}	6.76±0.14^{aA}	42.6±1.0^{aB}	nd	42.6±1.2^{aB}	6.76±0.12^{aA}
	24h	SSF	58.3±1.1^{aB}	6.68±0.09^{aA}	35.0±1.3^{aA}	0.618±0.017^{b}	34.4±1.1^{aA}	6.68±0.09^{aA}

	48h		59.7±1.3^{aB}	6.60±0.18^{aA}	33.7±1.0^{aA}	0.448±0.016^{a}	33.3±1.3^{aA}	6.61±0.13^{aA}
***Liquorilactobacillus uvarum* No. 245**	24h	SMF	47.6±0.9^{aA}	6.95±0.14^{aA}	45.4±1.2^{aB}	nd	45.4±1.1^{aB}	6.95±0.13^{aA}
	48h		48.1±1.0^{aA}	6.77±0.15^{aA}	45.0±1.1^{aB}	nd	45.0±1.3^{aB}	6.77±0.15^{aA}
	24h	SSF	54.4±1.3^{aB}	6.90±0.13^{aA}	38.7±1.1^{aA}	0.444±0.014^{b}	38.3±1.2^{aA}	6.90±0.14^{aA}
	48h		54.8±1.1^{aB}	6.86±0.14^{aA}	38.4±1.3^{aA}	0.399±0.016^{a}	38.0±1.4^{aA}	6.86±0.12^{aA}

Control (I) - Spirulina powder and water mixture. 1:20 w/w; Control (II) - Spirulina powder and water mixture. 1:2 w/w; SSF – solid state fermentation; SMF – submerged fermentation; SFA - saturated fatty acids; MUFA - monounstaturated fatty acids; PUFA - polyunsaturated fatty acids; omega 3 - omega 3 fatty acids; omega 6 - omega 6 fatty acids; omega 9 - omega 9 fatty acids; nd - not determined. Data are represented as means (n = 3) ± standard error. $^{a\text{-}b}$ mean values denoted with different letters. indicates significantly different values between the different duration (24 and 48 h) of fermentation ($p \leq 0.05$); $^{A\text{-}C}$ mean values denoted with different letters indicates significantly different values between samples fermented with the same strain at different conditions ($p \leq 0.05$).
